# Using innovative antimicrobial glove technology to reduce the risk of surgical wound contamination following glove perforation

**DOI:** 10.1186/1753-6561-5-S6-O33

**Published:** 2011-06-29

**Authors:** CE Edmiston, CJ Krepel, BD Lewis, KR Brown, PJ Rossi, GR Seabrook, G Daeschlein

**Affiliations:** 1 Surgery, Medical College of Wisconsin, Milwaukee, Wisconsin, USA; 2 Ernst Moritz Amdt University, Greifswald, Germany

## Introduction / objectives

Surgical gloves create a protective barrier, however high perforation rates (6%>60%) are reported in the literature. A recent study has suggested linkage between glove perforation and increased risk of SSI. This investigation evaluated a model of microbial passage through conventional single (A), double-thickness (B) and a tri-layer innovative surgical glove (C) with antimicrobial activity.

## Methods

Bacterial passage was assessed following multiple glove puncture using *S. aureus* and *B.* (*Pseudomonas*) *diminuta* (BD) in a model of gross wound contamination in volunteers in Groups A, B and C. Using microbiological methods bacterial passage was assessed at 5, 10, 30 and 45 minute exposure, expressed as cfu per unit time. A total of 6 repetitions were made for each glove/time interval***.*** The Mann Whitney test was used to assess the differences in microbial passage between the three groups.

## Results

Microbial passage was evaluated separately (5, 10, 30 and 45 min) and combined (5/10 and 30/45). No significant differences were observed in microbial passage between Groups A and B at 10, 30, or 45 minutes, a significant difference was observed in Group C at 5, 30 and 45 minutes compared to A and B for SA and BD. When timed groups were combine a significant reduction in passage of SA and BD was observed compared to Groups A and B.

## Conclusion

An antimicrobial surgical glove was effective at reducing microbial passage (*p*<*0.05-p*<*0.005*) following glove perforation compared to single or double-layer gloves. These findings suggest further studies are warranted to assess the clinical efficacy of an innovative antimicrobial glove technology as a SSI risk reduction strategy.

## Disclosure of interest

None declared.

